# Identifying Lesbian, Gay, Bisexual, and Transgender Search Terminology: A Systematic Review of Health Systematic Reviews

**DOI:** 10.1371/journal.pone.0156210

**Published:** 2016-05-24

**Authors:** Joseph G. L. Lee, Thomas Ylioja, Mellanye Lackey

**Affiliations:** 1 Department of Health Education and Promotion, College of Health and Human Performance, East Carolina University, Greenville, North Carolina, United States of America; 2 School of Social Work, University of Pittsburgh, Pittsburgh, Pennsylvania, United States of America; 3 Eccles Health Sciences Library, University of Utah, Salt Lake City, Utah, United States of America; Institute of Tropical Medicine (NEKKEN), Nagasaki University, JAPAN

## Abstract

Research on the health of lesbian, gay, bisexual, and transgender (LGBT) populations can provide important information to address existing health inequalities. Finding existing research in LGBT health can prove challenging due to the plethora of terminology used. We sought to describe existing search strategies and to identify more comprehensive LGBT search terminology. We iteratively created a search string to identify systematic reviews and meta-analyses about LGBT health and implemented it in Embase, PubMed/MEDLINE, and PsycINFO databases on May 28–29, 2015. We hand-searched the journal *LGBT Health*. Inclusion criteria were: systematic reviews and meta-analyses that addressed LGBT health, used systematic searching, and used independent coders for inclusion. The published search terminology in each record and search strings provided by authors on request were cross-referenced with our original search to identify additional terminology. Our search process identified 19 systematic reviews meeting inclusion criteria. The number of search terms used to identify LGBT-related records ranged from 1 to 31. From the included studies, we identified 46 new search terms related to LGBT health. We removed five search terms as inappropriate and added five search terms used in the field. The resulting search string included 82 terms. There is room to improve the quality of searching and reporting in LGBT health systematic reviews. Future work should attempt to enhance the positive predictive value of LGBT health searches. Our findings can assist LGBT health reviewers in capturing the diversity of LGBT terminology when searching.

## Introduction

The historical invisibility of lesbian, gay, bisexual, and transgender (LGBT) lives is something of a pattern in LGBT health research, driven by invisibility in public health surveillance systems [1]. Nonetheless, a growing number of high-quality data sources have documented health inequalities in chronic disease, infectious disease, mental health, and violent victimization [2]. A growing research agenda seeks to examine the origins of these inequalities and evaluate interventions to address them [2]. As the body of LGBT health research grows, evidence synthesis through reproducible systematic review and meta-analysis methodologies becomes increasingly important [3].

Systematic reviews provide a rigorous approach to identifying existing literature thereby limiting bias through the selection of studies [3]. Additionally, systematic reviews and meta-analyses can show trends across multiple smaller studies that are individually difficult to interpret given their small size [3]. Searches of the grey literature (i.e., unpublished in academic journals) can help counteract the effect of publication bias [4]. Systematic reviews and meta-analyses can inform evidence-based interventions and identify practice-based evidence from community organizations [5]. Systematic reviews are particularly important when study results are spread across multiple disciplines and academic as well as non-academic journals.

To achieve these important goals, however, systematic reviews and meta-analyses must be conducted in a high-quality manner [6]. In the initial stages of identifying the existing literature through a systematic search process, bias can be introduced by failing to identify relevant studies. Implementing high-quality searches and reporting them according to the Preferred Reporting Items for Systematic Reviews and Meta-Analyses (PRISMA) guidelines remains a challenge for systematic reviews in general [7, 8].

Although the importance of systematic review and meta-analysis methodologies and reporting are of general concern to health researchers, the incredible diversity of terminology used to describe and define LGBT communities by researchers, advocates, and community members provides an additional challenge to systematically reviewing LGBT health literature [9, 10]. To assist LGBT health researchers in the literature search process, we sought to examine the keyword searches used, report on the searches, and propose additional terminology for use in LGBT health searches. We operationalized this in two aims: (1) to describe characteristics of search strategies used in LGBT health systematic reviews and (2) to identify a comprehensive set of LGBT search terms that can be used to increase the sensitivity of LGBT health systematic review searches.

## Methods

### Search

Using PubMed/MEDLINE, we developed keywords and MeSH terms in two domains (systematic reviews and homosexuality). We based our starting search keywords on resources from the University of Texas Libraries [11] for systematic review terminology and our previous work reviewing the literature on tobacco interventions for LGBT populations [12]. After iteratively testing and improving our search strings, we then translated our search into the controlled vocabulary of other databases. We excluded certain unrelated terms because their abbreviations are used in LGBT health, for example: “markov state model” and “men who have sex with men” are both abbreviated MSM. A matrix of controlled vocabulary and individual database search strings are reported in S1 File. We implemented our search on May 28–29, 2015, in three health databases: Embase, PsycINFO, and PubMed/MEDLINE. We hand-searched the Web site of the journal *LGBT Health* on September 11, 2015. We set no date, geographic, or language limits in our search or in our inclusion process. Our final PubMed/MEDLINE search string was:

((bisexual[tiab] OR bisexuality[MeSH Terms] OR bisexuality[tiab] OR bisexuals[tiab] OR gay[tiab] OR gays[tiab] OR GLB[tiab] OR GLBT[tiab] OR homosexual[tiab] OR homosexualities[tiab] OR homosexuality[MeSH Terms] OR homosexuality[tiab] OR homosexuals[tiab] OR intersex[tiab] OR lesbian[tiab] OR lesbianism[tiab] OR lesbians[tiab] OR LGB[tiab] OR LGBT[tiab] OR "men who have sex with men"[tiab] OR msm[tiab] OR queer[tiab] OR "sexual minorities"[tiab] OR "sexual minority"[tiab] OR "sexual orientation"[tiab] OR transgender[tiab] OR transgendered[tiab] OR transgenders[tiab] OR transsexual[tiab] OR transsexualism[MeSH Terms] OR transsexualism[tiab] OR transsexuality[tiab] OR transsexuals[tiab] OR "women loving women"[tiab] OR "women who have sex with women"[tiab] OR WSW[tiab]) NOT (gay[au] OR "laparoscopic gastric bypass"[tiab] OR "markov state model" OR "multiple source method"[tiab]))

AND

(systematic*[tiab] AND (bibliographic*[tiab] OR literature[tiab] OR review[tiab] OR reviewed[tiab] OR reviews[tiab])) OR (comprehensive*[tiab] AND (bibliographic*[tiab] OR literature[tiab])) OR "integrative literature review"[tiab] OR "integrative research review"[tiab] OR "integrative review"[tiab] OR “research synthesis”[tiab] OR “research integration”[tiab] OR meta-analys*[tiab] OR meta-analyz*[tiab] OR meta-analyt*[tiab] OR metaanalys*[tiab] OR metaanalyz*[tiab] OR metaanalyt*[tiab] OR “meta-analysis as topic”[MeSH:noexp] OR Meta-Analysis[ptyp] OR ((review[tiab] AND (rationale[tiab] OR evidence[tiab])) AND review[pt])

### Inclusion

We set our criteria for inclusion as being a systematic review related to LGBT health. We defined systematic review as (a) using a set of keywords in (b) two or more databases with (c) independent coders assessing all identified records for inclusion or exclusion. Guidelines from Agency for Healthcare Research and Quality (AHRQ) (recommendation 7.6.6) [13], Cochrane (recommendation 7.2.4) [14], and the U.S. Institute of Medicine (IOM; recommendation 3.3.3) [15] all recommend dual independent coding for inclusion to reduce error and increase confidence in the findings.

In defining LGBT health, we sought to include studies that addressed domains such as injury prevention, chronic disease, mental health, violence, and sexual health and well-being. We a priori excluded: (a) HIV/AIDS-specific studies (because they often focus exclusively on same-sex behavior and we wished to focus this search on a broader definition of LGBT health), (b) studies about same-sex contact and resulting risk for HIV and sexually transmitted infections, (c) studies about the impact of LGBT parents on children (because the children may not be LGBT), (d) studies about treatment of homosexuality or gender dysphoria (including hormone therapy), and (e) studies about the origins of homosexuality.

**Fig 1:**
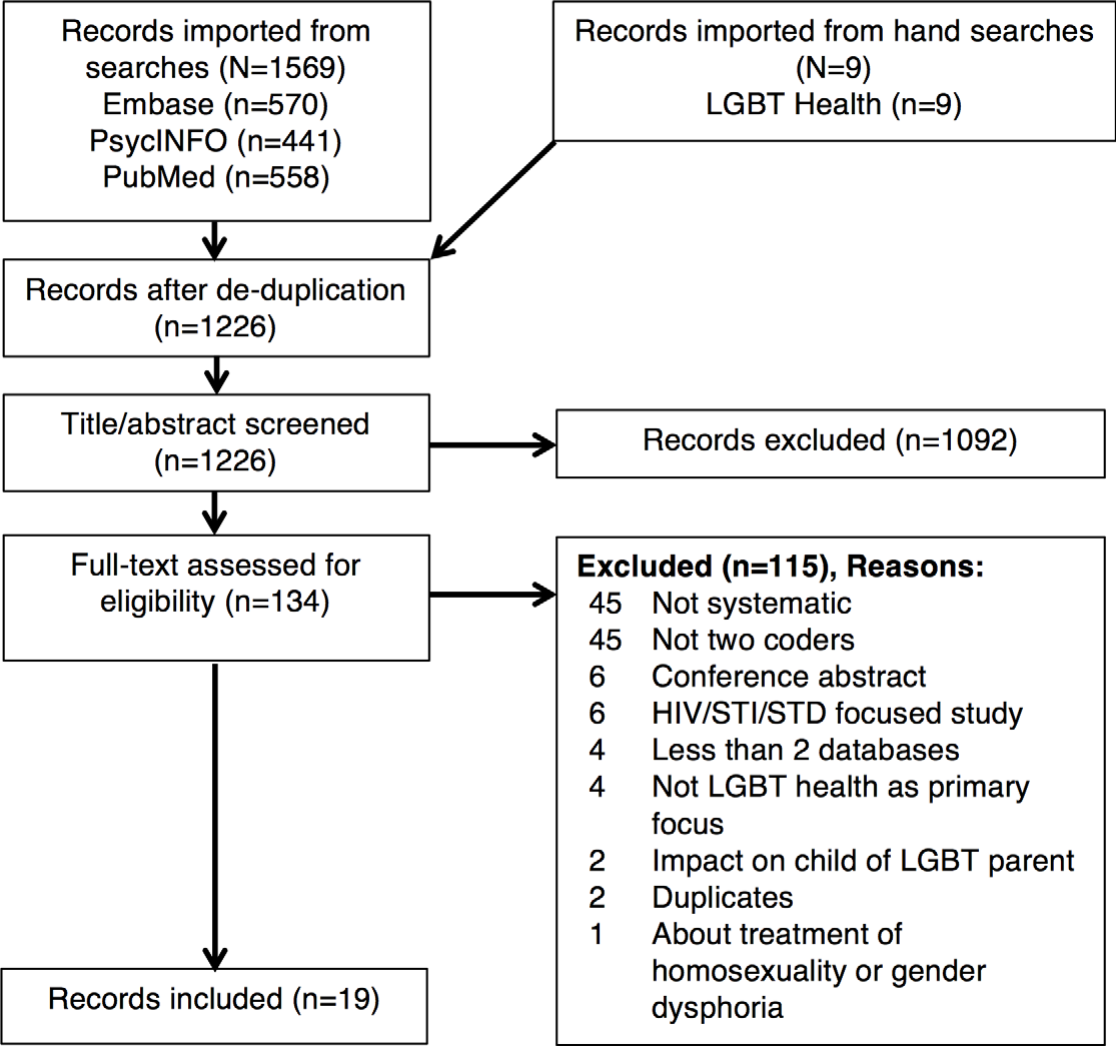
PRISMA Flow Diagram, May 28–29, 2015.

After de-duplication, two authors independently screened the title and abstract of 1,226 records for potential inclusion or exclusion, removing studies clearly not related to the research question. Two authors then independently screened each of the 134 full text records identified for possible inclusion. At each stage, differences in coding were reconciled through discussion and consensus of at least two authors. We did not calculate reliability because we viewed the goal of independent coders being one of enhancing sensitivity to eligible records rather than one of establishing uniformity. We used Covidence (covidence.org) to manage the screening and coding process. Fig 1 shows the inclusion process.

### Abstraction

Two authors independently abstracted the following information from the included records: (a) if the review reported a search string in keeping with the PRISMA guideline #8 (“Present full electronic search strategy for at least one database, including any limits used, such that it could be repeated”) [16], (b) the keywords used to define the LGBT population of interest, (c) the databases searched, (d) any hand-searched journals, (e) inclusion of grey literature, (f) if the authors reviewed reference lists of included studies, (g) involvement of a librarian (because inclusion of a librarian has been shown to improve search quality [17]), (h) if the study assessed publication bias, (i) whether the study included meta-analysis, and (j) the area of LGBT health covered. We discussed any discrepancies in extraction coding and obtained consensus among authors, then exported the data into an evidence table.

In the interest of assembling maximum data on search terms used for LGBT health, we e-mailed the corresponding author to request the full search string if it was not reported in the manuscript. We then cross-referenced the abstracted search strategies with our own search strategy to create the most comprehensive search string for LGBT health systematic reviews.

## Results

We identified 19 studies meeting our inclusion criteria. These studies examined aging [18, 19], alcohol use [20], breast cancer [21], cardiovascular outcomes for transgender users of sex steroids [22], health information–seeking behaviors [23], intimate partner violence [24–26], mental health [27], stressful childhood experiences [28], substance abuse [29, 30], suicide [31], tobacco use [12, 32, 33], and victimization/abuse [34].

Review terminology and databases are reported in Table 1. In accordance with PRISMA reporting guidelines for searches, 13 presented a final search string from a specific database, including any limits. The number of LGBT-related keywords ranged from 1 [24, 31] to 31 [35]. One study reported conflicting information about what databases were searched [31]. For the remaining 18 studies, the number of databases ranged from 2 [24, 30, 34], the minimum required for study inclusion, to 15 [18]. The most commonly used databases in the 19 identified studies were PubMed/MEDLINE (17 studies), PsycINFO (15 studies), CINAHL (8 studies), Web of Science/Knowledge (7 studies), and Embase (6 studies). Nine studies searched the grey literature, 17 reported searching reference lists, and 6 reported involvement of a librarian as an author in the methods or in the acknowledgements. Six of the 19 included studies assessed publication bias. Nine studies conducted a meta-analysis. Table 2 reports the search characteristics.

**Table 1:** Study databases and search keywords (or final search strategy, if reported), N = 19, May 28–29, 2015.

Study	N: Databases	Page: Search or Keywords
Badenes-Ribera *et al*., 2015 [24]	2: PubMed and PsycINFO	p. 47: Lesbian
Batejan *et al*., 2015 [35]	4: PsycINFO, Medline, SocINDEX, and ERIC	Appendix: bicurious OR bisexual(s) OR bisexuality OR gay(s) OR GLB OR GLBQ OR GLBs OR GLBT OR GLBTQ OR heteroflexible OR homosexual(s) OR homosexuality OR lesbian(s) OR LGB OR LGBQ OR LGBS OR LGBT OR LGBT OR lesbigay OR men who have sex with men OR MSM OR queer(s) OR same sex attracted OR same sex attracted youth OR SSA OR SSAY OR same-sex relations OR sexual minority OR sexual orientation OR women who have sex with women OR WSW
Blosnich *et al*., 2013 [32]	10: Academic Search Elite, Alt HealthWatch, CAB Abstracts 1990-Present, CINAHL with Full Text, Health Source Consumer Edition, Health Source: Nursing/Academic Edition, MEDLINE, PsycARTICLES, PsycINFO and Social Work Abstracts	p. 67: ((homosexual* OR gay OR ‘sexual minority’ OR ‘sexual minorities’ OR lesbian* OR bisexual* OR queer OR ‘sexual orientation’ OR ‘men who have sex with men’ OR MSM OR ‘women who have sex with women’ OR WSW)
Buller *et al*., 2014 [25]	13: MEDLINE, EMBASE, Global Health, PsycINFO, the Health Management Information Consortium database [HMIC], Social Policy and Practice, the Cumulative Index to Nursing and Allied Health Literature [CINAHL], the International Bibliography of the Social Sciences [IBSS], Web of Science, Africa Web, Index Medicus for South-East Asia Region [IMSEAR], Index Medicus for the Eastern Mediterranean Region [IMEMR], and Latin American and Caribbean Health Sciences Literature [LILACS])	In S1 File (PubMed via OVID): Homosexuality/ OR Homosexuality, Male/ OR Transsexualism/ OR Bisexuality/ OR Homosexual*.mp. OR Transexual*.mp. OR Bisexual*.mp. OR Transgender.mp. OR (MSM or men who have sex with men or ((man or men or male*) adj3 (gay or homosexual or queer or bisexual* or transsexual* or transgender)) or LGBT).mp.
Elamin *et al*., 2010 [22]	5: Ovid MEDLINE, Ovid Embase, Ovid PsycInfo, Thomson Scientific Web of Science and Elsevier Scopus	Upon Request: 1. (trans adj (sexual$ or gender$ or male or men or women or female or people or person$)).mp. [mp = title, original title, abstract, name of substance word, subject heading word] 2. gender identity/ and su.fs. 3. sex reversal, gonadal/ 4. ((sex$ or gender) adj (transition$ or transform$ or reassign$ or chang$)).mp. [mp = title, original title, abstract, name of substance word, subject heading word] 5. transsexualism/ or (trans adj sexual$).mp. or transexual$.mp. or transsexual$.mp. [mp = title, original title, abstract, name of substance word, subject heading word] 6. ((gender or sexual$) adj2 (dysphor$ or identity)).mp. [mp = title, original title, abstract, name of substance word, subject heading word] 7. (crossgender or (cross adj (sex$ or gender$))).mp. [mp = title, original title, abstract, name of substance word, subject heading word] 8. (transgender$ or (trans adj gender$)).mp. [mp = title, original title, abstract, name of substance word, subject heading word] 9. (m2f or f2m or "male-to-female" or "female-to-male").mp. and (1 or 2 or 3 or 4 or 5 or 6 or 7 or 8) [mp = title, original title, abstract, name of substance word, subject heading word] 10. or/1-9 11. limit 10 to humans
Finkenauer *et al*., 2012 [18]	15: CINAHL (1942-), Medline (1942-), Health Services/Technology Assessment Texts, Web of Science, EMBASE (1947-), Sociological Abstracts (1952), Social Services Abstracts (1806-), Gender Studies Database (1972-), LGBT Life with Full Text, Ageline (1978-), PsycINFO (1806-), Scopus, ERIC, The New York Academy of Medicine Grey Literature Report, and Dissertations & Theses: Full Text.	p. 313: transgender or transsexual or transexual or transman or transwoman or genderqueer or “gender queer” or LGBT or GLBT or transvestite or crossdress or “cross dress ” or “cross-dress ” or “drag queen” or “drag queens” or “drag king” or “drag kings” or “gender identity disorder” or “gender dysphori ”
Friedman *et al*., 2011 [34]	2: MEDLINE and PsycINFO	P. 1482: NR but example keywords include gay, lesbian, bisexual, sexual orientation, homosexual, and homosexuality
Goldbach *et al*., 2014 [29]	3: PsychINFO, PubMED, and EBSCO.	p. 351: lesbian OR gay OR bisexual OR sexual minority
Harding *et al*., 2012 [19]	4: Medline (1950-present), PsycINFO (1806–2010), Cinahl (1982–2010), and ASSIA (1987–2010).	p. 603: homosexual OR lesbian OR gay OR transgender OR bisexual
King *et al*., 2008 [27]	12: Medline, Embase, PsycINFO, Cinahl, the Cochrane Library Database, the Web of Knowledge, the Applied Social Sciences Index and Abstracts, the International Bibliography of the Social Sciences, Sociological Abstracts, the Campbell Collaboration and grey literature databases for Google and Google Scholar	NR
Langhinrichsen-Rohling *et al*., 2012 [26]	7: Academic Search Premier, Education Resources Information Center, Medical Literature Analysis and Retrieval System Online, PsycINFO, CINAHL, Biomedical Reference Collection, and SocINDEX	p. 205: N/A (authors manually selected LGBT-related studies from a search on intimate partner violence)
Lee *et al*., 2009 [33]	7: Seven databases were searched for peer-reviewed research articles (Cumulative Index to Nursing and Allied Health Literature (CINAHL), Cochrane Library via Wiley InterScience, Education Resources Information Center (ERIC), Health Source: Nursing/Academic, Institute for Scientific Information (ISI) Web of Science, PsycINFO via EBSCO Host and PubMed)	p. 282: homosexuality OR homosexual OR gay OR "sexual minority" OR "female homosexuality" OR "homosexuality, female" OR lesbian OR bisexuality OR bisexual OR transgender OR transsexual OR transsexualism OR transsexuality OR MSM OR queer OR "sexual orientation" OR "men who have sex with men” OR WSW OR “women loving women” OR “women who have sex with women” OR lesbianism
Lee *et al*., 2014 [12]	8: Cochrane Central Register of Controlled Trials via Wiley Online Library; Cumulative Index to Nursing and Allied Health Literature (CINAHL), Global Health, PsycINFO, and Social Work Abstracts via EBSCO; Embase; PubMed; and Scopus.	p. 824: (homosexuality[MeSH Terms] OR homosexuality[tiab] OR homosexual[tiab] OR gay[tiab] OR LGBT[tiab] OR GLBT[tiab] OR LGB[tiab] OR “sexual minority”[tiab] OR “sexual minorities”[tiab] OR lesbian[tiab] OR bisexuality[MeSH Terms] OR bisexuality[tiab] OR bisexual[tiab] OR transsexualism[MeSH Terms] OR transsexualism[tiab] OR transgender[tiab] OR transsexual[tiab] OR trans- sexuality[tiab] OR msm[tiab] OR queer[tiab] OR “sexual orientation”[tiab] OR “men who have sex with men”[tiab] OR WSW[tiab] OR “women loving women”[tiab] OR “women who have sex with women”[tiab] OR lesbianism[tiab])
Liu *et al*., 2014 [20]	3: PubMed, WanFang Data, Google Scholar	p. 2: “MSM” OR “men who have sex with men” OR “gay” OR “homosexual”
Marshal *et al*., 2008 [30]	2: PsycINFO and Medline	p. 548: NR but example keywords include: gay, lesbian, bisexual, LGB
Meads *et al*., 2013 [21]	8: Cochrane library (CDSR, CENTRAL, HTA, DARE, NHSEED), MEDLINE, EMBASE, PsycINFO, CAB abstracts, Web of Science (SCI, SSCI), SIGLE, Social Care Online	p. 2: lesbian, gay women, queer, bisexual, sexual preference, sexual orientation
Pompili *et al*., 2014 [31]	Unknown: MedLine, Excerpta Medica, PsycLit and PsycINFO, and Index Medicus reported in methods section; in results 3 are reported as being searched: PubMed, Scopus, and Web of Knowledge.	p. 1904: Bisexuality
Rose *et al*., 2013 [23]	4: MEDLINE, Applied Social Services Index and Abstracts, Sociological Abstracts and Social Service Abstracts.	p. 419: lesbian OR gay OR bisexual
Schneeberger *et al*., 2014 [28]	5: MEDLINE (Ovid), PubMed, Web of Science, Google Scholar, and PsycNet (includes PsycINFO, PsycBOOKS, PsycARTICLES, PsycTESTS),	p. 2–3: lesbian, gay, bisexual, transgender, transsexual, homosexual, men who have sex with men

Note: NR = Not Reported

**Table 2:** Study Characteristics, N = 19, May 28–29, 2015.

Study	Replicable String Reported	Reported Librarian Involvement	Searched Citations	Grey Literature Search	Hand Search	Assessed Publication Bias	Meta-analysis	Research Area
Badenes-Ribera *et al*., 2015 [24]	Yes	No	Yes	No	Yes*	No	Yes	Intimate Partner Violence
Batejan *et al*., 2015 [35]	No	No	Yes	Yes	No	Yes	Yes	Self-Harm, Non-Suicidal
Blosnich *et al*., 2013 [32]	Yes	No	Yes	No	No	No	No	Tobacco
Buller *et al*., 2014 [25]	Yes	No	Yes	No	Yes**	Yes	Yes	Intimate Partner Violence
Elamin *et al*., 2010 [22]	No	Yes	Yes	Yes	No	No	Yes	Cardiovascular Disease, Sex Steroids
Finkenauer *et al*., 2012 [18]	Yes	Yes	Yes	Yes	No***	No	No	Aging
Friedman *et al*., 2011 [34]	No	No	Yes	Yes	No	Yes	Yes	Victimization and Abuse
Goldbach *et al*., 2014 [29]	Yes	No	Yes	No	No	Yes	Yes	Substance Abuse
Harding *et al*., 2012 [19]	Yes	No	Yes	No	No	No	No	Aging
King *et al*., 2008 [27]	No	Yes	Yes	Yes	No	No	Yes	Mental Health
Langhinrichsen-Rohling *et al*., 2012 [26]	No	No	Yes	No	No	No	No	Intimate Partner Violence
Lee *et al*., 2009 [33]	Yes	Yes	Yes	No	No	No	No	Tobacco
Lee *et al*., 2014 [12]	Yes	Yes	No	Yes	No	No	No	Tobacco
Liu *et al*., 2014 [20]	Yes	Yes	Yes	No	No	Yes	Yes	Alcohol
Marshal *et al*., 2008 [30]	No	No	Yes	Yes	No	Yes	Yes	Substance Abuse
Meads *et al*., 2013 [21]	Yes	No	Yes	Yes	No	No	No	Breast Cancer
Pompili *et al*., 2014 [31]	Yes	No	Yes	No	No	No	No	Suicide
Rose *et al*., 2013 [23]	Yes	No	No	No	No	No	No	Health Information Seeking Behaviors
Schneeberger *et al*., 2014 [28]	Yes	No	Yes	Yes	No	No	No	Stressful Childhood Experiences

* Hand search: Journal of Homosexuality; Journal of Lesbian Studies; Journal of Gay & Lesbian Social Service; Journal of GLBT Family Studies; Journal of LGBT Health Research; Journal of LGBT Issues in Counseling

** Hand search: Journal of Homosexuality, Journal of Gay & Lesbian Social Services, and Journal of LGBT Issues in Counseling

*** But, completed a "manual search of published reference material"

Our initial PubMed/MEDLINE search contained 36 search terms. Cross-referencing these with the identified search terms, we identified an additional 46 LGBT-related terms. We excluded three of these—“cross dress,” “drag king(s),” and “drag queen(s)”—used in a review on transgender aging [18] because these terms do not necessarily indicate a sexual orientation or gender identity [10]. We excluded two additional terms, the abbreviations “SSA” and “SSAY” (for same-sex attracted [youth]), because these picked up thousands of un-related articles, leaving 41 new terms. We added five additional terms that were not used in any search. These are terms we have seen used in LGBT health research: “same gender loving” [36], “same sex couple” [37], “same sex couples” [38], “sexual and gender minority” [39], and its plural version, “sexual and gender minorities.” This full list of 82 terms is presented below with bolded terms coming from the identified reviews and italicized terms added based on their use in the field.

**(bicurious[tiab] OR** bisexual[tiab] OR bisexuality[MeSH Terms] OR bisexuality[tiab] OR bisexuals[tiab] OR **“cross sex”[tiab] OR crossgender[tiab] OR F2M[tiab] OR “female-to-male”[tiab] OR** gay[tiab] OR gays[tiab] OR **“gender change”[tiab] OR “gender dysphoria”[tiab] OR “gender identity”[tiab] OR “gender queer”[tiab] OR “gender reassign”[tiab] OR “gender transform”[tiab] OR “gender transition”[tiab] OR genderqueer[tiab] OR** GLB[tiab] OR **GLBQ[tiab] OR GLBs[tiab] OR** GLBT[tiab] OR **GLBTQ [tiab] OR heteroflexible [tiab] OR** homosexual[tiab] OR homosexualities[tiab] OR homosexuality[MeSH Terms] OR homosexuality[tiab] OR homosexuals[tiab] OR intersex[tiab] OR lesbian[tiab] OR lesbianism[tiab] OR lesbians[tiab] OR **lesbigay[tiab] OR** LGB[tiab] OR **LGBQ[tiab] OR LGBS[tiab] OR** LGBT[tiab] OR **M2F[tiab] OR “male-to-female”[tiab] OR** “men who have sex with men”[tiab] OR msm[tiab] OR queer[tiab] OR *“same gender loving”[tiab] OR* **“same sex attracted”[tiab] OR** *“same sex couple”[tiab] OR “same sex couples”[tiab] OR* **“same sex relations”[tiab] OR “sex change”[tiab] OR “sex reassign”[tiab] OR “sex reversal”[tiab] OR “sex transform”[tiab] OR “sex transition”[tiab] OR** *“sexual and gender minorities”[tiab] OR “sexual and gender minority”[tiab] OR* **“sexual identity”[tiab] OR** “sexual minorities”[tiab] OR “sexual minority”[tiab] OR “sexual orientation”[tiab] OR **“sexual preference”[tiab] OR “trans female”[tiab] OR “trans male”[tiab] OR “trans man”[tiab] OR “trans men”[tiab] OR “trans people”[tiab] OR “trans person”[tiab] OR “trans woman”[tiab] OR “trans-sexuality”[tiab] OR transexual[tiab] OR** transgender[tiab] OR transgendered[tiab] OR transgenders[tiab] OR transsexual[tiab] OR transsexualism[MeSH Terms] OR transsexualism[tiab] OR transsexuality[tiab] OR transsexuals[tiab] OR **transvestite[tiab] OR** “women loving women”[tiab] OR “women who have sex with women”[tiab] OR WSW[tiab] NOT ("laparoscopic gastric bypass"[tiab] OR gay[au] OR "markov state model" OR "multiple source method"[tiab]))

The body of evidence identified from the LGBT search domain in PubMed/MEDLINE on November 4, 2015, is 40,759 and 53,451 for our original and the expanded search, respectively.

## Discussion

There is room for improvement in the implementation and reporting of literature searches in LGBT health systematic reviews and meta-analyses. Strong evidence synthesis is essential to address a multitude of health concerns for LGBT populations. Authors have an ethical obligation to the field to reduce bias from study identification to ensure limited available resources are used effectively.

A strong evidence base for documenting, understanding, and intervening on LGBT health inequalities requires high-quality systematic reviews and meta-analyses. Comprehensive guidelines are available from AHRQ [13], Cochrane [14], and IOM [15]. Based on this assessment of the state of LGBT health systematic reviews, we recommend that authors of systematic reviews in LGBT health use and report (and peer reviewers hold to account): (a) including a librarian or information specialist as collaborator to improve the search quality [17], (b) using more than one academic database, (c) using the controlled vocabulary of databases, (d) conducting searches of the reference lists of included studies, (e) reporting a complete specific search string so that the review can be updated as new literature emerges, (f) using dual coders for inclusion to improve data quality, and (g) using dual coders for abstraction or, at minimum, a reviewer to confirm and validate evidence tables [40]. The work presented in this paper contributes to the development of better searches given the complex terminology used in LGBT health [9], but each of these recommendations on its own would contribute to stronger evidence synthesis in the field of LGBT health.

There are important limitations to this study. First, we used a somewhat restrictive definition of systematic review requiring dual, independent coding of titles and abstracts. Although AHRQ [13], Cochrane [14], and IOM [15] recommend dual independent coding for inclusion, many systematic reviews—some with strong search strategies—were ineligible due to not reporting the number of coders or having a single author decide which papers to include. Second, we did not empirically test our comprehensive search against other strings used by each of the studies identified in our search, thus we cannot be certain to what extent our search would improve the identification of relevant studies. We viewed this as being an unfair comparison because the identification of studies is a multi-step process that is unique to the aims of a given study. Third, searches must balance sensitivity and specificity; our work represents a preliminary effort to address search coverage by increasing sensitivity to LGBT health-related articles. Further work is needed to ensure a balance between sensitive searches and more specific searches. Fourth, changes in terminology to define and describe LGBT populations are likely already happening [9]; although our work provides a thorough list of keywords for searching, future reviewers should consider the ever-shifting landscape of LGBT terminology. Fifth, we conducted our original search in three academic databases; searching a larger number of databases could have resulted in inclusion of additional reviews. Sixth, we did not assess the role of publication bias in our identification of search terminology; results could be influenced by unpublished reviews that may have poorly designed search strings.

The lives of LGBT individuals have historically been invisible in health data [1] and in popular culture [41]. With growing research to address health inequalities, it is imperative that rigorous methods to identify and synthesize existing research be employed. With diverse and shifting terminology being used, researchers should carefully consider the terminology used to identify as much of the relevant literature as possible. Efforts to combat health inequalities are only as strong as the evidence available to know what inequalities exist, how they come into being, and how to intervene against them.

## Supporting Information

S1 File Systematic review protocol. (DOCX)
