# Supplementary material for: Identifying Lesbian, Gay, Bisexual, and Transgender Search Terminology: A Systematic Review of Health Systematic Reviews
Source: PLoS One. 2016 May 24;11(5):e0156210. doi: 10.1371/journal.pone.0156210 (PMC4878791; doi:10.1371/journal.pone.0156210)
Supplement: S1 File — (DOCX) [file pone.0156210.s001.docx]

Improving lesbian, gay, bisexual, and transgender systematic review search terminology: a systematic review of systematic reviews

Joseph G. L. Lee, PhD, MPH

- develop project, help code, draft manuscript

Thomas Ylioja, MSW

- develop project, help code, edit manuscript

Mellanye Lackey, MSI

- develop project, conduct searches, help code, edit manuscript

Department of Health Education and Promotion, College of Health and Human Performance, East Carolina University, Greenville, NC

School of Social Work, University of Pittsburgh

Eccles Health Sciences Library, University of Utah, Salt Lake City, UT USA

**Research Question**: How can the field improve LGBT health research synthesis by improving literature searches?

Aim_1_: Identify the use of LGBT health keywords in LGBT health systematic reviews and recommend an optimal group of keywords.

Protocol

1. Develop search string
2. Implement with "evidence synthesis" and "systematic review" keywords
3. Conduct in **PubMed**, **Embase**, and **PsycINFO** databases
   1. Hand search, LGBT Health
   2. (point is not to find all reviews, just to find a good cross-section to review terminology)
4. Identify articles that are systematic reviews or meta-analyses
   1. Population: Systematic reviews and meta-analyses about LGBT health issues
      1. Must be systematic, i.e., have two reviewers independently assess records for inclusion or exclusion [and use more than one database added 2015-09-28]
      2. Must include one or more domain of sexual orientation (identity, behavior, attraction)
         1. Must be a primary focus of the paper, not just an ancillary analysis, identified moderator, or subpopulation
      3. No geography restrictions
      4. ~~English language only~~ *(removed 2015-08-11)*
      5. Limits of health topics
         1. Not HIV/AIDS-specific studies
         2. Not PLW HIV/AIDS-specific populations
         3. Not same-sex contact and resulting STI/STD/HIV risk studies (e.g., MSM as a risk group without a focus on identity; anal dysplasia screening among MSM would be excluded but an GB anal dysplasia screening intervention review would be included); a study about intimate partner violence that used MSM as it's measure of sexual orientation would be included.
         4. Not studies of impact of LGB parents on children
         5. Not studies about treatment of homosexuality or transgender as a disease
         6. Not studies about origins of homosexuality
         7. Not about treatment of gender dysphoria, hormone therapies, or gender reassignment surgery
   2. [added 2015-09-30] Order of operations for exclusion in full-text review and reconciliation of divergent coding:
      1. Is the topic LGBT health? IF NO, exclude as NOT LGBT HEALTH.
      2. Is the paper a conference abstract? IF YES, exclude as ABSTRACT.
      3. Is the review not systematic because it is a critical, comprehensive, or author-knowledge-based review with no attempt to utilize standardized database searching coding, etc. IF YES, exclude as NOT SYSTEMATIC
      4. Does the review pass step iii but is focused on HIV, STDs, and STIs? IF YES, exclude as HIV/STD FOCUSED
      5. Does the review search only one database? IF YES, exclude as ONE DATABASE
      6. Does the review have dual independent coding of titles/abstracts? IF **NO**, exclude as NOT TWO CODERS.
   3. Outcome: LGBT-related search terminology used in search
   4. Time: N/A
   5. Setting: Global
5. Abstract LGBT-related search terminology used
   1. Code % of articles not reporting a replicable string. See example here.^1^ Note that PRISMA statement says: "Present full electronic search strategy for at least one database, including any limits used, such that it could be repeated." (#8).^2^
      1. Request search terms from authors, if not available
6. Code for databases used
7. Code for meta-analysis
8. ~~Code for random-effects meta-analysis~~ *(removed 2015-08-11)*
9. ~~Code for use of opposite effects proportion~~ *(removed 2015-08-11)*
10. Code for gray literature search *(added 2015-08-11)*
11. Code for involvement of a librarian in the search process *(added 2015-08-11)*
12. Code for outcome type (e.g., tobacco, alcohol, etc.)
13. Using all identified strings, create complete string and iteratively test what we think is an optimal string.
14. Apply LGBT-related search terminology in PubMed
    1. Identify string coverage of current universe (of our full search of all LGBT keywords).
    2. If feasible: Implement authors' strings and % difference in LGBT coverage
15. Describe string coverage at single point in time (now).
16. Report words commonly used, omitted
17. Suggest optimal strings for LGBT, LGB, LB, GB, B
18. Note updates, changes to this protocol by date

**Search Strategy Development**

**Table**. Search Development in PubMed for Identifying Systematic Reviews

| Date, n | Sexual & Gender Minority | Review | Content Areas |
| --- | --- | --- | --- |
| 2014-10-21, 3649 | (homosexuality[MeSH Terms] OR homosexuality[tiab] OR homosexual[tiab] OR gay[tiab] OR LGBT[tiab] OR GLBT[tiab] OR LGB[tiab] OR "sexual minority"[tiab] OR "sexual minorities"[tiab] OR lesbian[tiab] OR bisexuality[MeSH Terms] OR bisexuality[tiab] OR bisexual[tiab] OR transsexualism[MeSH Terms] OR transsexualism[tiab] OR transgender[tiab] OR transsexual[tiab] OR transsexuality[tiab] OR msm[tiab] OR queer[tiab] OR "sexual orientation"[tiab] OR "men who have sex with men"[tiab] OR WSW[tiab] OR "women loving women"[tiab] OR "women who have sex with women"[tiab] OR lesbianism[tiab]) | ("meta-analysis"[PT] OR "meta-analysis"[TIAB] OR "review"[PT] OR "systematic review"[TIAB]) |  |
| 2014-10-21, 1534 | + NOT ((AIDS[TIAB] OR HIV[TIAB])))) NOT "laparoscopic gastric bypass" | "" |  |
| 2014-10-21, 841 | "" | + 2000:2014[DP] |  |
| 2014-10-21, 663 | "" | alt: 2004:2014[DP] |  |
| 2014-10-21, 840 | NOT gay[au] | "" |  |
| 2014-12-18, 1555 | (("homosexuality"[MeSH Terms] OR homosexuality[tiab] OR homosexual[tiab] OR gay[tiab] OR LGBT[tiab] OR GLBT[tiab] OR LGB[tiab] OR "sexual minority"[tiab] OR "sexual minorities"[tiab] OR lesbian[tiab] OR "bisexuality"[MeSH Terms] OR bisexuality[tiab] OR bisexual[tiab] OR "transsexualism"[MeSH Terms] OR transsexualism[tiab] OR transgender[tiab] OR transsexual[tiab] OR transsexuality[tiab] OR msm[tiab] OR queer[tiab] OR "sexual orientation"[tiab] OR "men who have sex with men"[tiab] OR WSW[tiab] OR "women loving women"[tiab] OR "women who have sex with women"[tiab] OR lesbianism[tiab]) AND ("meta-analysis"[PT] OR "meta-analysis"[TIAB] OR "review"[PT] OR "systematic review"[TIAB])) NOT (AIDS[TIAB] OR HIV[TIAB] OR "laparoscopic gastric bypass"[tiab] OR gay[au]) |  |  |
| 2014-12-18, 684 | + date range: | 2004:2014[DP] |  |
| 2014-12-18, 93 | ok | ok | ("smoking"[MeSH Terms] OR "smoking"[TIAB] OR "tobacco"[MeSH Terms] OR "tobacco"[TIAB] OR "tobacco products"[MeSH Terms] OR "tobacco products"[TIAB]) OR ATOD[TIAB] OR "other drugs"[TIAB] OR meth[TIAB] OR methamphetamine[tiab] OR methamphetamine[MeSH Terms] OR "cannabis"[MeSH Terms] OR "cannabis"[TIAB] OR "marijuana"[TIAB] OR "suicide"[MeSH Terms] OR "suicide"[TIAB] OR "self-harm"[TIAB] OR "suicidal ideation"[TIAB] OR "violence"[MeSH Terms] OR "violence"[TIAB]) OR "crime victims"[MeSH Terms] OR "crime victims"[TIAB] OR "victimization"[TIAB] OR "bullying"[MeSH Terms] OR "bullying"[TIAB]) |
| 2014-12-18, 112 | ok | ok | ("smoking"[MeSH Terms] OR "smoking cessation"[MESH] OR "tobacco use disorder"[MESH] OR "tobacco use cessation"[MESH] OR "tobacco use"[MESH] OR "smoking"[TIAB] OR "tobacco"[MeSH Terms] OR "tobacco"[TIAB] OR "tobacco products"[MeSH Terms] OR "tobacco products"[TIAB]) OR ATOD[TIAB] OR "substance-related disorders"[MESH] OR "substance abuse treatment centers"[MESH] OR "substance abuse"[tiab] OR "other drugs"[TIAB] OR meth[TIAB] OR methamphetamine[tiab] OR methamphetamine[MeSH Terms] OR "cannabis"[MeSH Terms] OR "cannabis"[TIAB] OR "marijuana"[TIAB] OR "suicide"[MeSH Terms] OR "suicide"[TIAB] OR "self-harm"[TIAB] OR "suicidal ideation"[TIAB] OR "violence"[MeSH Terms] OR "violence"[TIAB]) OR "crime victims"[MeSH Terms] OR "crime victims"[TIAB] OR "victimization"[TIAB] OR "bullying"[MeSH Terms] OR "bullying"[TIAB]) |
| 2014-12-18, 118 |  |  | ("smoking"[MeSH Terms] OR "smoking cessation"[MESH] OR "tobacco use disorder"[MESH] OR "tobacco use cessation"[MESH] OR "tobacco use"[MESH] OR "smoking"[TIAB] OR "tobacco"[MeSH Terms] OR "tobacco"[TIAB] OR "tobacco products"[MeSH Terms] OR "tobacco products"[TIAB]) OR ATOD[TIAB] OR "substance-related disorders"[MESH] OR "substance abuse treatment centers"[MESH] OR "substance abuse"[tiab] OR alcohol[TIAB] OR "alcohol-induced disorders"[MESH] OR "alcohol-related disorders"[MESH] OR "alcohol drinking"[MESH] OR alcoholism[MESH] OR "other drugs"[TIAB] OR meth[TIAB] OR methamphetamine[tiab] OR methamphetamine[MeSH Terms] OR "cannabis"[MeSH Terms] OR "cannabis"[TIAB] OR "marijuana"[TIAB] OR "suicide"[MeSH Terms] OR "suicide"[TIAB] OR "self-harm"[TIAB] OR "suicidal ideation"[TIAB] OR "violence"[MeSH Terms] OR "violence"[TIAB]) OR "crime victims"[MeSH Terms] OR "crime victims"[TIAB] OR "victimization"[TIAB] OR "bullying"[MeSH Terms] OR "bullying"[TIAB]) |
| 2014-12-18 | Add in GLB:  (("homosexuality"[MeSH Terms] OR homosexuality[tiab] OR homosexual[tiab] OR gay[tiab] OR LGBT[tiab] OR GLBT[tiab] OR LGB[tiab] OR GLB[tiab] OR "sexual minority"[tiab] OR "sexual minorities"[tiab] OR lesbian[tiab] OR "bisexuality"[MeSH Terms] OR bisexuality[tiab] OR bisexual[tiab] OR "transsexualism"[MeSH Terms] OR transsexualism[tiab] OR transgender[tiab] OR transsexual[tiab] OR transsexuality[tiab] OR msm[tiab] OR queer[tiab] OR "sexual orientation"[tiab] OR "men who have sex with men"[tiab] OR WSW[tiab] OR "women loving women"[tiab] OR "women who have sex with women"[tiab] OR lesbianism[tiab]) NOT (AIDS[TIAB] OR HIV[TIAB] OR "laparoscopic gastric bypass"[tiab] OR gay[au]) | ("meta-analysis"[PT] OR "meta-analysis"[TIAB] OR "review"[PT] OR "systematic review"[TIAB]) |  |
| 2014-12-18, 121 |  |  | (("smoking"[MeSH Terms] OR "smoking cessation"[MESH] OR "tobacco use disorder"[MESH] OR "tobacco use cessation"[MESH] OR "tobacco use"[MESH] OR "smoking"[TIAB] OR "tobacco"[MeSH Terms] OR "tobacco"[TIAB] OR "tobacco products"[MeSH Terms] OR "tobacco products"[TIAB]) OR ATOD[TIAB] OR "substance-related disorders"[MESH] OR "substance abuse treatment centers"[MESH] OR "substance abuse"[tiab] OR alcohol[TIAB] OR "alcohol-induced disorders"[MESH] OR "alcohol-related disorders"[MESH] OR "alcohol drinking"[MESH] OR "alcoholism"[MeSH Terms] OR "other drugs"[TIAB] OR meth[TIAB] OR methamphetamine[tiab] OR "methamphetamine"[MeSH Terms] OR "cannabis"[MeSH Terms] OR "cannabis"[TIAB] OR "marijuana"[TIAB] OR cocaine[TIAB] OR ecstasy[TIAB] OR "suicide"[MeSH Terms] OR "suicide"[TIAB] OR "self-harm"[TIAB] OR "suicidal ideation"[TIAB] OR "violence"[MeSH Terms] OR "violence"[TIAB]) OR "crime victims"[MeSH Terms] OR "crime victims"[TIAB] OR "victimization"[TIAB] OR assault[TIAB] OR "hate crime"[tiab] OR "bullying"[MeSH Terms] OR "bullying"[TIAB] |
| (("smoking"[MeSH Terms] OR "smoking cessation"[MESH] OR "tobacco use disorder"[MESH] OR "tobacco use cessation"[MESH] OR "tobacco use"[MESH] OR "smoking"[TIAB] OR "tobacco"[MeSH Terms] OR "tobacco"[TIAB] OR "tobacco products"[MeSH Terms] OR "tobacco products"[TIAB] OR ATOD[TIAB] OR "substance-related disorders"[MESH] OR "substance abuse treatment centers"[MESH] OR "substance abuse"[tiab] OR alcohol[TIAB] OR "alcohol-induced disorders"[MESH] OR "alcohol-related disorders"[MESH] OR "alcohol drinking"[MESH] OR "alcoholism"[MeSH Terms] OR "other drugs"[TIAB] OR meth[TIAB] OR methamphetamine[tiab] OR "methamphetamine"[MeSH Terms] OR "cannabis"[MeSH Terms] OR "cannabis"[TIAB] OR "marijuana"[TIAB] OR cocaine[TIAB] OR ecstasy[TIAB] OR "suicide"[MeSH Terms] OR "suicide"[TIAB] OR "self-harm"[TIAB] OR "suicidal ideation"[TIAB] OR "violence"[MeSH Terms] OR "violence"[TIAB]) OR "crime victims"[MeSH Terms] OR "crime victims"[TIAB] OR "victimization"[TIAB] OR assault[TIAB] OR "hate crime"[tiab] OR "bullying"[MeSH Terms] OR "bullying"[TIAB])  AND  ("meta-analysis"[PT] OR "meta-analysis"[TIAB] OR "review"[PT] OR "systematic review"[TIAB])  AND  2004[PDAT] : 2015[PDAT]  AND  (("homosexuality"[MeSH Terms] OR homosexuality[tiab] OR homosexual[tiab] OR gay[tiab] OR LGBT[tiab] OR GLBT[tiab] OR LGB[tiab] OR GLB[tiab] OR "sexual minority"[tiab] OR "sexual minorities"[tiab] OR lesbian[tiab] OR "bisexuality"[MeSH Terms] OR bisexuality[tiab] OR bisexual[tiab] OR "transsexualism"[MeSH Terms] OR transsexualism[tiab] OR transgender[tiab] OR transsexual[tiab] OR transsexuality[tiab] OR msm[tiab] OR queer[tiab] OR "sexual orientation"[tiab] OR "men who have sex with men"[tiab] OR WSW[tiab] OR "women loving women"[tiab] OR "women who have sex with women"[tiab] OR lesbianism[tiab]) NOT ("laparoscopic gastric bypass"[tiab] OR gay[au]))  2015-03-27: Gives n = 246 (removed AIDS/HIV restriction, updated year) | | | |
| 2015-05-07, extra LGBT keywords from homosexuality entry terms  N=784 | (("homosexuality"[MeSH Terms] OR homosexuality[tiab] OR homosexual[tiab] Homosexuals[tiab] OR homosexualities[tiab] OR gay[tiab] OR gays[tiab] OR transgendered[tiab] OR transgenders[tiab] OR intersex[tiab] OR transsexuals[tiab] OR bisexuals[tiab]OR LGBT[tiab] OR GLBT[tiab] OR LGB[tiab] OR GLB[tiab] OR "sexual minority"[tiab] OR "sexual minorities"[tiab] OR lesbian[tiab] OR lesbians[tiab] OR "bisexuality"[MeSH Terms] OR bisexuality[tiab] OR bisexual[tiab] OR "transsexualism"[MeSH Terms] OR transsexualism[tiab] OR transgender[tiab] OR transsexual[tiab] OR transsexuality[tiab] OR msm[tiab] OR queer[tiab] OR "sexual orientation"[tiab] OR "men who have sex with men"[tiab] OR WSW[tiab] OR "women loving women"[tiab] OR "women who have sex with women"[tiab] OR lesbianism[tiab]) NOT ("laparoscopic gastric bypass"[tiab] OR gay[au]) | ("meta-analysis"[PT] OR "meta-analysis"[TIAB] OR "review"[PT] OR "systematic review"[TIAB]) |  |
| 2015-05-12, search improvements^[[1]](#footnote-1)^ on phone, n = 485  2015-05-26, N=492 (then fixed missing OR), 2015-05-28 N=558  FINAL PUBMED | (("homosexuality"[MeSH Terms] OR homosexuality[tiab] OR homosexual[tiab] OR Homosexuals[tiab] OR homosexualities[tiab] OR gay[tiab] OR gays[tiab] OR transgendered[tiab] OR transgenders[tiab] OR intersex[tiab] OR transsexuals[tiab] OR bisexuals[tiab]OR LGBT[tiab] OR GLBT[tiab] OR LGB[tiab] OR GLB[tiab] OR "sexual minority"[tiab] OR "sexual minorities"[tiab] OR lesbian[tiab] OR lesbians[tiab] OR "bisexuality"[MeSH Terms] OR bisexuality[tiab] OR bisexual[tiab] OR "transsexualism"[MeSH Terms] OR transsexualism[tiab] OR transgender[tiab] OR transsexual[tiab] OR transsexuality[tiab] OR msm[tiab] OR queer[tiab] OR "sexual orientation"[tiab] OR "men who have sex with men"[tiab] OR WSW[tiab] OR "women loving women"[tiab] OR "women who have sex with women"[tiab] OR lesbianism[tiab]) NOT ("laparoscopic gastric bypass"[tiab] OR gay[au] OR "markov state model" OR "multiple source method"[tiab])) | (systematic*[tiab] AND (bibliographic*[TIAB] OR literature[tiab] OR review[tiab] OR reviewed[tiab] OR reviews[tiab])) OR  (comprehensive*[TIAB] AND (bibliographic*[TIAB] OR literature[tiab])) OR  "integrative literature review"[tiab]  OR"integrative research review"[tiab] OR  "integrative review"[tiab] OR  “research synthesis”[tiab] OR “research integration”[tiab] OR meta-analys*[tiab] OR meta-analyz*[tiab] OR meta-analyt*[tiab] OR metaanalys*[tiab] OR metaanalyz*[tiab] OR metaanalyt*[tiab] OR “meta-analysis as topic”[MeSH:noexp] OR Meta-Analysis[ptyp] OR ((review[tiab] AND (rationale[tiab] OR evidence[tiab])) AND review[pt]) |  |

**Conversion of Controlled Vocabulary**

| **PubMed/MeSH** | **Embase/Emtree** | **PsycINFO/Subjects** |
| --- | --- | --- |
| homosexuality | **""** | **""** |
| bisexuality | **""** | **""** |
| transsexualism | **""** | **""** |
| meta-analysis as topic | meta analysis | Meta Analysis |

**PsycINFO Search 2015-05-29, N=441**

(DE (homosexuality OR bisexuality OR transexualism) OR TI (homosexuality OR homosexuality OR homosexual OR Homosexuals OR homosexualities OR gay OR gays OR transgendered OR transgenders OR intersex OR transsexuals OR bisexuals OR LGBT OR GLBT OR LGB OR GLB OR "sexual minority" OR "sexual minorities" OR lesbian OR lesbians OR "bisexuality" OR bisexuality OR bisexual OR "transsexualism" OR transsexualism OR transgender OR transsexual OR transsexuality OR msm OR queer OR "sexual orientation" OR "men who have sex with men" OR WSW OR "women loving women" OR "women who have sex with women" OR lesbianism) NOT ("laparoscopic gastric bypass" OR gay OR "markov state model" OR "multiple source method")) OR AB (homosexuality OR homosexuality OR homosexual Homosexuals OR homosexualities OR gay OR gays OR transgendered OR transgenders OR intersex OR transsexuals OR bisexuals OR LGBT OR GLBT OR LGB OR GLB OR "sexual minority" OR "sexual minorities" OR lesbian OR lesbians OR "bisexuality" OR bisexuality OR bisexual OR "transsexualism" OR transsexualism OR transgender OR transsexual OR transsexuality OR msm OR queer OR "sexual orientation" OR "men who have sex with men" OR WSW OR "women loving women" OR "women who have sex with women" OR lesbianism) NOT ("laparoscopic gastric bypass" OR "markov state model" OR "multiple source method")))

**AND**

((DE (meta analysis) OR TI ((systematic* AND (bibliographic* OR literature OR review OR reviewed OR reviews)) OR (comprehensive* AND (bibliographic* OR literature)) OR  "integrative literature review" OR "integrative research review" OR  "integrative review" OR  “research synthesis” OR “research integration” OR meta-analys* OR meta-analyz* OR meta-analyt* OR metaanalys* OR metaanalyz* OR metaanalyt* OR ((review AND (rationale OR evidence)))) OR AB ((systematic* AND (bibliographic* OR literature OR review OR reviewed OR reviews))OR  (comprehensive*[TIAB] AND (bibliographic*[TIAB] OR literature[tiab])) OR  "integrative literature review"  OR"integrative research review" OR  "integrative review" OR  “research synthesis” OR “research integration” OR meta-analys* OR meta-analyz* OR meta-analyt* OR metaanalys* OR metaanalyz* OR metaanalyt* OR ((review AND (rationale OR evidence)))))

Then selected peer reviewed only.


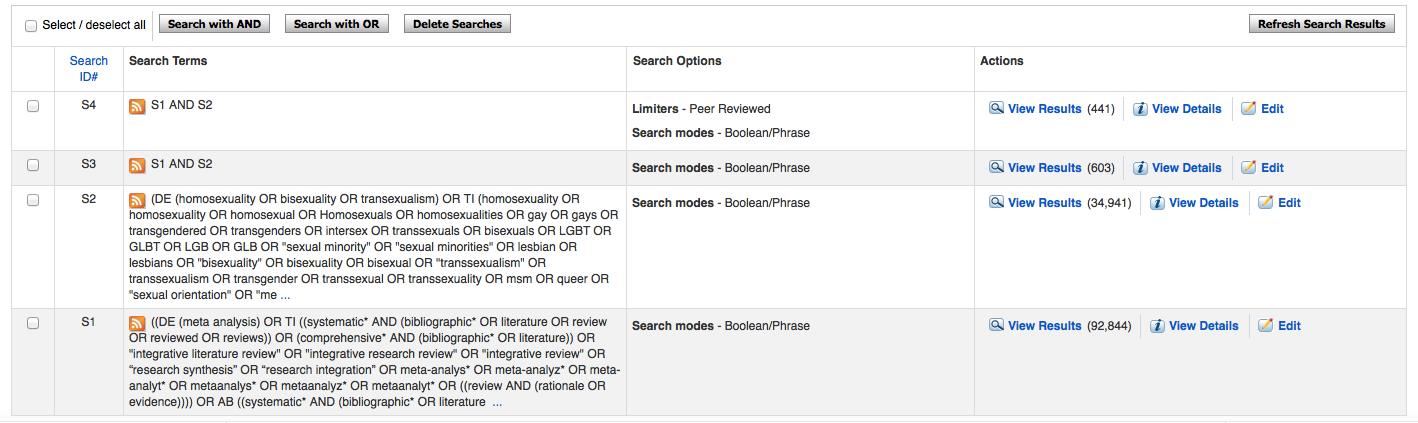


**Embase Search, 2015-05-29, N=570**

Note: Embase uses a field called ‘other terms’ to hold minor subject terms. Classifying minor or major varies by indexer, and I found many SRs that didn’t have systematic review as a major term. So, when I search for only __/exp OR __:ab,ti , it leaves out results that are actually systematic reviews. It misses them, because it’s not searching the :ot field. If I leave off the field tag, then it does. I vote for leaving it off in this case. If it seems like the results are off, I will revisit. - ML

**('systematic review'** OR **'systematic review'**/exp OR **'systematic review (topic)'** OR **'systematic review (topic)'**/exp OR **'bibliographic review'** OR **'systematic literature review'** OR **'integrative literature review'** OR **'integrative research review'** OR **'research synthesis'** OR **'research integration'** OR **'meta analysis'** OR **'meta analysis'**/exp OR **'meta analysis (topic)'** OR **'meta analysis (topic)'**/exp)

AND

**('homosexuality'**/exp OR **homosexuality** OR **'homosexual'**/exp OR **homosexual** OR **'homosexuals'**/exp OR **homosexuals** OR **homosexualities** OR **gay** OR **'gays'**/exp OR **gays** OR **transgendered** OR **'transgenders'**/exp OR **transgenders** OR **'intersex'**/exp OR **intersex** OR **'transsexuals'**/exp OR **transsexuals** OR **'bisexuals'**/exp OR **bisexuals** OR **lgbt** OR **glbt** OR **'lgb'**/exp OR **lgb** OR **glb** OR **'sexual minority'**/exp OR **'sexual minority'** OR **'sexual minorities'**/exp OR **'sexual minorities'** OR **'lesbian'**/exp OR **lesbian** OR **'lesbians'**/exp OR **lesbians** OR **'bisexuality'**/exp OR **bisexuality** OR **'bisexual'**/exp OR **bisexual** OR **'transsexualism'**/exp OR **transsexualism** OR **'transgender'**/exp OR **transgender** OR **'transsexual'**/exp OR **transsexual** OR **'transsexuality'**/exp OR **transsexuality** OR **msm** OR **queer** OR **'sexual orientation'**/exp OR **'sexual orientation'** OR **'men who have sex with men'**/exp OR **'men who have sex with men'** OR **wsw** OR **'women loving women'** OR **'women who have sex with women'**/exp OR **'women who have sex with women'** OR **'lesbianism'**/exp OR **lesbianism** NOT (**'laparoscopic gastric bypass'** OR **gay:au** OR **'markov state model'** OR **'multiple source method'**))

AND

AND [embase]/lim

**
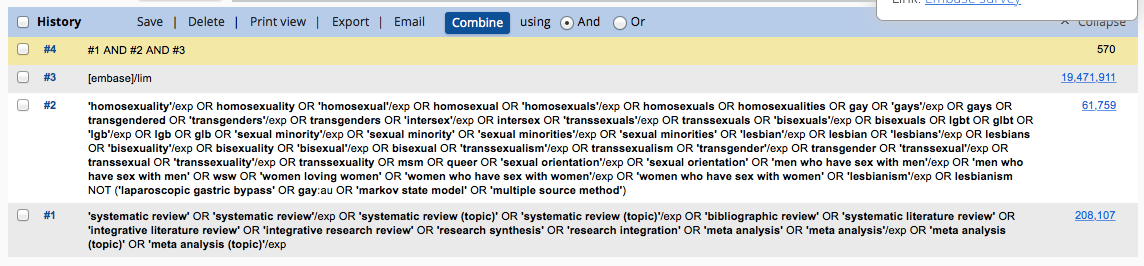
**

**PubMed Search 2015-05-28, N=558 (note from table above)**

**
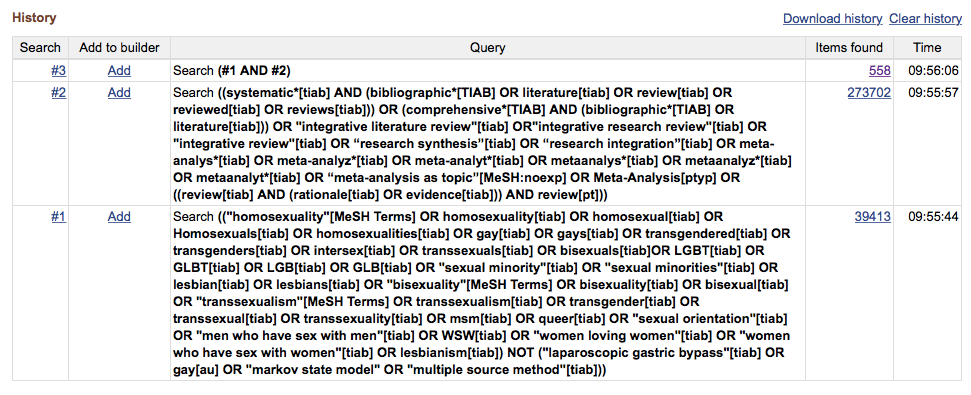
**

**Draft Abstraction Form**

Article ID

First Author Last Name

INCLUSION

No date limit

Is a *systematic* review or meta-analysis?

Is related to LGBT health other than with a primary focus on HIV/AIDS or STIs?

Is about attraction, behavior, or identity of sexual orientation or about gender identity?

ABSTRACTION

Evidence Table

| Author, Year: LGBT Population | Health Outcome | Databases | LGBT Portion of Search String | Potentially relevant papers identified by meta-string and not by original string |
| --- | --- | --- | --- | --- |
|  |  |  |  |  |

Note: * = meta-analysis

**References**

- 1. 1. Yoshii A, Plaut DA, McGraw KA, Anderson MJ, Wellik KE. Analysis of the reporting of search strategies in Cochrane systematic reviews. *Journal of the Medical Library Association : JMLA.* 2009;97(1):21-29.
  2. 2. Moher D, Liberati A, Tetzlaff J, Altman DG. Preferred reporting items for systematic reviews and meta-analyses: the PRISMA statement. *PLoS Med.* 2009;6(7):e1000097.

1. From: http://libguides.sph.uth.tmc.edu/pubmed_filters [↑](#footnote-ref-1)
